# Research Strategies for Low-Survival Cancers

**DOI:** 10.3390/cancers13030528

**Published:** 2021-01-30

**Authors:** Caroline Conway, Denis M. Collins, Amanda McCann, Kellie Dean

**Affiliations:** 1 Genomics Core Facility, Biomedical Sciences Research Institute, Ulster University, Coleraine BT52 1SA, UK; c.conway@ulster.ac.uk; 2 National Institute for Cellular Biotechnology, Dublin City University, Glasnevin, Dublin 9, Ireland; denis.collins@dcu.ie; 3 UCD Conway Institute of Biomolecular and Biomedical Research and UCD School of Medicine College of Health and Agricultural Science (CHAS), University College Dublin, Belfield, Dublin 4, Ireland; amanda.mccann@ucd.ie; 4 School of Biochemistry and Cell Biology, 3.91 Western Gateway Building, University College Cork, Cork T12 XF62, Ireland

**Keywords:** low survival cancer, immuno-oncology, insulin signaling, chromatin re-modelling, transforming growth factor-beta signaling

## Abstract

**Simple Summary:**

The Irish Association for Cancer Research (IACR) held its 56th annual conference from 26–28 February 2020, in Galway, Ireland. This report provides a summary overview of the work presented at the conference, which had a particular focus on low-survival cancers. There is a clinical need for new and improved treatment strategies for low-survival cancers. Dynamic and insightful pre-clinical research programs are a critical component in addressing this need, but challenges exist. This manuscript reports on the novel research strategies currently being investigated to improve outcomes for patients with poor prognosis cancers.

**Abstract:**

While substantial progress has been made to improve the diagnosis, prognosis, and survivorship of patients with cancer, certain cancer types, along with metastatic and refractory disease, remain clinical challenges. To improve patient outcomes, ultimately, the cancer research community must meet and overcome these challenges, leading to improved approaches to treat the most difficult cancers. Here, we discuss research progress aimed at gaining a better understanding of the molecular and cellular changes in tumor cells and the surrounding stroma, presented at the 56th Irish Association for Cancer Research (IACR) Annual Conference. With a focus on poor prognosis cancers, such as esophageal and chemo-resistant colorectal cancers, we highlight how detailed molecular knowledge of tumor and stromal biology can provide windows of opportunity for biomarker discovery and therapeutic targets. Even with previously characterized targets, such as phosphoinositide 3-kinase (PI3K), one of the most altered proteins in all human cancers, new insights into how this protein may be more effectively inhibited through novel combination therapies is presented.

## 1. Introduction

Advances in treatment and detection have seen overall cancer 5-year survival rates improve by 18% in Ireland from 1994 to 2014 (adults 15–99, all cancers excluding non-melanoma skin cancer, male and female, unstandardized for age) (National Cancer Registry, Ireland). However, there are some cancers for which survival rates have remained stubbornly low. The definition of low-survival cancers can be variable but can generally be described as cancers with a 5-year survival rate of <50%. Using this cut-off in Ireland, this includes pancreatic (7.4%), ovarian and uterine adnexal (38.1%), stomach (26%), esophageal (21%), lung (15.4%), brain (21.8%) liver (16.5%), biliary (14.5%), hypo-pharyngeal (33.3%), multiple myeloma (45.8%), and nasal cavity/middle ear/accessory sinus cancers (49.6%) (National Cancer Registry, Ireland; all statistics are for adults aged 15–99, male and female, combined except for sex specific cancers; all data is non-age standardized. All values represent % of 5-year survival rates). Despite expected differences in healthcare systems, the composition of this list remains relatively consistent across multiple high-income jurisdictions, including Australia, Canada, Denmark, Ireland, New Zealand, the UK, and Norway [1]. It should also be stated that the survival rates for low-survival cancers have not remained static. Arnold et al. reported that, over a 20-year period (1995–2014), there were significant improvements in survival rates for patients <75 years at diagnosis versus those >75 years and that these changes were also notable for poor prognosis cancers, such as esophageal, stomach, pancreatic, and lung cancers [1]. Improvements in cancer control (defined by the authors as “increased survival, decreased mortality and incidence”) were observed in stomach, colon, lung (for males) and ovarian cancers. Survival outcomes can also vary greatly within cancer types based on the clinical staging at diagnosis. For instance, the overall 5-year survival rate for colorectal cancer in Ireland is 61.3%. Stage I and II colon cancer has 5-year survival rates of 97.3% and 87.2% but drops to 10.3% for Stage IV disease (National Cancer Registry, Ireland). This data is mirrored in multiple countries (https://gco.iarc.fr/ International Agency for Research on Cancer). Within cancers, there are also subtypes that can vary in their survival outcomes. Molecular profiling of breast tumors has shown that patients with hormone receptor-positive breast cancers (Luminal A/Luminal B) have better survival outcomes than those with hormone receptor negative cancers (triple negative and human epidermal growth factor receptor 2 (HER2)-enriched molecular subtypes) [2]. Low-survival cancers present multiple research challenges. Late-stage diagnosis generally means a limited window for clinical trial opportunities. No surgical intervention means no tissue for in-depth research or molecular pathology epidemiological studies as examples. Rare tumors can often fall into the low-survival category for this reason. Therefore, there is an obvious, unmet, clinical need for improved treatment strategies for low-survival cancers, and the cancer research community is responding. We report on data presented at the 56th Irish Association for Cancer Research (IACR) Annual Conference, covering research on metastatic colon cancer, head and neck squamous cell carcinoma (HNSCC), esophageal cancer, and ovarian and pancreatic cancers. 

## 2. Gaining Insights into Esophageal and Colorectal Cancers Using a Molecular Pathology Epidemiological Approach

Molecular pathology epidemiology (MPE) studies investigate the interaction between epidemiological risk factors in relation to cancer risk or survival, according to the molecular characteristics or subtypes of that tumor. Professor Helen Coleman, Lead of the Cancer Epidemiology Research Group at the Centre for Public Health at Queen’s University Belfast and Deputy Director of the Northern Ireland Cancer Registry, outlined her group’s current research using MPE studies, conducted at Queen’s University Belfast, which provide insights into esophageal and colorectal neoplasia. Northern Ireland is uniquely well placed to conduct research of this study design, given the infrastructure offered by the population-based Northern Ireland Cancer Registry, Northern Ireland Biobank, and the affiliated Precision Medicine Centre at Queen’s University Belfast [3,4], along with the possibility of anonymized data linkages between these organizations. Professor Coleman outlined two major MPE initiatives benefitting from this infrastructure. The first interdisciplinary and collaborative MPE initiative established a data and tissue resource of almost 700 Stage II and III colon cancer patients. This resource has been utilized to investigate the associations between medication use, such as aspirin and statins, in relation to colon cancer survival, according to strata of biomarkers involved in their metabolism [5,6]. For example, aspirin use has been associated with improved survival in patients with high prostaglandin-endoperoxide synthase 2 (PTGS2) expression, but not phosphatidylinositol-4,5-bisphosphate 3-kinase catalytic subunit alpha (PI3KCA) mutational status [6]. MPE studies require large sample sizes and with that bring methodological challenges for scoring immunohistochemical biomarkers in a high throughput manner. The team at Belfast benefitted greatly from the parallel innovation of QuPath technology, which was developed and validated using some of the data collected in this colon cancer cohort [7,8].

Applying MPE methods to cancers of poor prognosis, such as esophageal cancer, raises significant challenges. This is a rarer tumor and often presents at a late stage, which precludes surgical resection and therefore the opportunity to collect tissue on a sufficiently large scale. However, the hypotheses could still hold true that lifestyle factors may influence patient survival according to molecular characteristics of the tumor. Small studies conducted by Professor Coleman and collaborators suggest, for example, that alcohol intake and vitamin D receptor expression are associated with survival in esophageal adenocarcinoma patients [9,10]. As part of her Cancer Research UK Career Establishment Award, Professor Coleman is currently working with the UK-wide Oesophageal Cancer Clinical and Molecular Stratification (OCCAMS) consortium and the Precision Medicine Centre at Belfast to establish a tissue microarray cohort within their cohort of over 3000 patients. This will enable MPE and other biomarker research questions to be investigated in esophageal neoplasia in the future. 

## 3. Lack of Efficacy of Immunotherapy for Colorectal Cancer Patients: Influence of Intestinal Stromal Cells and the Inflammatory Tumor Microenvironment

Immunotherapies for the treatment of cancer, which activate endogenous anti-tumor immune responses, have revolutionized the field of oncology and shown remarkable success in some cancers. Advances in the use of immunotherapy for colorectal cancer (CRC), the second most commonly diagnosed and deadly cancer worldwide, have been largely unsuccessful [11,12]. A small proportion of patients, <4% of the metastatic CRCs, with mismatch repair deficiency (dMMR), respond to immunotherapies [13,14,15]. The other 96% of CRC patients do not respond, indicating a gap in our understanding of how these tumors regulate immune responses necessary for efficacy of immunotherapies [16,17]. Understanding how the tumor microenvironment (TME) regulates immune cell infiltration and function is necessary to develop effective immunotherapeutic strategies and overcome resistance [18,19].

Dr Aideen Ryan, Foundation Research Lecturer in Tumor Immunology in the School of Medicine, Pharmacology, and Therapeutics at the National University of Ireland (NUI), Galway, presented work by her group that showed how the influence of intestinal stromal cells could partially explain the limited efficacy of immunotherapies in CRC. Stromal cells of mesenchymal origin reside below the epithelial compartment and provide structural support in the intestine [20]. These intestinal stromal cells interact with both the epithelial cell compartments and infiltrate hematopoietic immune cells. The importance of these cells in regulating immune homeostasis during inflammation is well recognized. However, little is known about their function and phenotype in the inflammatory tumor microenvironment.

Dr Ryan’s group recently published data to address the roles of stromal intestinal cells using a syngeneic, immunogenic model of CRC. Their work showed that tumor necrosis factor alpha (TNFα)-initiated inflammatory signaling in colorectal cancer cells selectively induced programmed death-ligand 1 (PD-L1) expression in stromal cells. Using PD-L1 knockdown and antibody-mediated approaches, it was shown that stromal cell PD-L1 potentiated enhanced immunosuppression, characterized by the inhibition of an activated cluster of differentiation 8, positive (CD8+), granzyme B-secreting, and T cells in vitro, and the inhibition of CD8+ effector cells was associated with enhanced tumor progression. Additionally, stromal cell immunosuppressive and tumor-promoting effects could be reversed with administration of an anti–PD-1 therapy in vivo. Stromal cell PD-L1 expression was validated in two cohorts of clinical samples, and PD-L1 induction on human stromal cells, in response to exposure to the inflammatory secretome from human colon cancer cells, was also observed, irrespective of microsatellite instability. Collectively, Dr Ryan’s data showed that tumor-associated stromal cells support T-cell suppression by PD-L1 induction, which is dependent on colon cancer inflammatory signaling. This reveals a key role of mesenchymal stromal cell PD-L1 in suppression of CD8+ anti-tumor immune responses and potentiation of CRC progression [21]. These discoveries highlight the importance of characterizing the nature of the microenvironment in different tumors to optimize immunotherapeutic targeting and responses. 

Leading on from this, Dr Ryan’s team went on to identify a role for post-translational glycosylation of proteins in modulation anti-tumor immunity [22,23]. Specifically, the group investigated if stromal cell sialylation contributes to enhanced immunosuppression in the tumor microenvironment in both myeloma [22] and colorectal cancer [23]. In the context of CRC, using a mouse model, the team characterized a sialic acid profile of tumor-associated mesenchymal stromal cells (MSCs) exposed to tumor cell secretome, using lectin-based flow cytometry. Results from this work showed that sialic acid expression increased on MSCs exposed to the tumor cell secretome. Dr Ryan’s team observed that inflammatory tumor-conditioned MSCs significantly suppressed activation and proliferation of a cluster of differentiation 4, positive (CD4+) and CD8+ T-cells. Inhibition of sialic acid indicated that this effect was dependent on sialylation and reversed tumor-induced MSC immunosuppression. Dr Ryan indicated that their results demonstrate that non-hematopoietic stromal cells in the tumor-microenvironment express high levels of sialic acid, contributing to their ability to suppress activated T-effector cells. Understanding how glycosylation of stromal cells, and, more specifically, sialylation, is regulated and functions to enhance immunosuppression in the TME could uncover novel immune checkpoints to reactivate anti-tumor immunity.

## 4. TGF-β Signaling Responsiveness Influences to Therapeutic Response in Head and Neck Squamous Cell Carcinoma (HNSCC)

Therapeutic resistance is often intrinsic to the cancer cell, but less well appreciated is the role of the TME in determining resistance to genotoxic therapy. Factors in the TME can exert significant control of pathways whose execution is attributed to tumor cell intrinsic genome regulation. The largely underappreciated contribution of the TME to tumor cell intrinsic response to therapy underscores a gap that hampers cancer treatment optimization.

Transforming growth factor β (TGFβ) activity is highly limited in normal tissue but is pronounced in the extracellular compartment of the TME, where it facilitates immunosuppressive and pro-tumorigenic phenotypes of resident non-malignant cells. Cancer cells must escape TGFβ-mediated suppression of proliferation, yet many cancer cells remain transcriptionally responsive to TGFβ, indicative of a pro-tumorigenic benefit [24]. Moreover, radiation and chemotherapy induce TGFβ activation, implicating a TGFβ-rich TME in acquired resistance [25]. 

Professor Mary Helen Barcellos-Hoff, Department of Radiation Oncology, The University of California, San Francisco (UCSF), presented work from her group on human papillomavirus (HPV) associated HNSCC that provided new evidence of the role of TGFβ signaling in therapeutic response [26]. About one third of HNSCC recurs locally after standard care genotoxic therapy with radiation and cisplatin, underscoring the need to better understand the mechanisms by which tumors respond to genotoxic therapy [27]. HNSCC patients whose cancer is HPV-positive have a markedly better prognosis of 70% 5-year survival in response to standard of care, compared to 30% 5-year survival for those whose tumors are HPV-negative [28]. Professor Barcellos-Hoff presented data showing that HPV-positive cells are unresponsive to TGFβ, which compromises canonical DNA repair and shifts cells to use error-prone pathways that increase sensitivity to genotoxic chemotherapy or radiotherapy [26]. 

TGFβ controls execution of homologous recombination by indirectly regulating breast cancer gene 1 (BRCA1), whose message stability and translation is targeted by microRNA (miR)-182 [29]. HPV blockade of TGFβ, or inhibition using small molecule receptor kinase inhibitors in HNSCC cells, increases miR-182, which suppresses BRCA1 and thereby compromises homologous recombination [26]. Thus, TGFβ exerts profound control of homologous recombination by multiple mechanisms in HNSCC. Thus, tumors responsive to TGFβ-rich TME will be chemoradiation resistant, resulting in poor outcomes from standard of care.

## 5. Phosphoinositide 3-Kinase (PI3K)—New Insights into Effective Inhibition for Cancer Treatment

One of the highlights of the conference was the Irish Cancer Society Guest Lecture keynote presentation by Professor Lewis Cantley, the Meyer Director of the Sandra and Edward Meyer Cancer Center at Weill Cornell Medicine. Since his ground-breaking research on PI3K in the 1980s [30,31], Professor Cantley’s work has focused on how this enzyme influences many human diseases, including cancer. PI3K is activated by insulin and other growth factors to mediate cell growth. By converting the cell membrane lipid phosphatidylinositol 4,5-bisphosphate (PIP2) to phosphatidylinositol 3,4,5-triphosphate (PIP3), PI3K action activates multiple downstream targets, ultimately controlling fundamental cellular processes, such as metabolism, proliferation, growth, and survival [32]. 

Much of Cantley’s work stems from an intense desire to understand the molecular details of insulin signaling, not necessarily stopping cancer. However, the two are inextricably linked. 

The p110 alpha catalytic subunit of the PI3K enzyme, encoded by the *PIK3CA* gene, is one of the most frequently mutated oncogenes in human cancer, including breast, endometrial, colorectal, urinary tract, and ovarian cancer [33]. The gene is also amplified at high frequencies in HNSCC and gastric and cervical cancers [32]. Tumorigenic mutations of *PIK3CA* enhance the enzyme’s ability to be activated by the insulin receptor, leading to prolonged PIP3 production and keeping a downstream effector, AKT (protein kinase B, PKB), active for hours. Indeed, the mutational activation of the PI3K explains why most tumors consume glucose at high rates [34]. 

Due to hyperactivation of PI3K in multiple cancers, there have been robust efforts to inhibit PI3K for clinical benefit. During his presentation, Professor Cantley explained how the failures of early clinical trials with PI3K inhibitors resulted in his frustration regarding their ineffectiveness. Keeping in mind that this same enzyme mediates insulin responses in liver, muscle, fat, and other tissues, drugs that inhibit this enzyme have the expected effect of raising serum glucose and insulin levels [34]. This led to the hypothesis that insulin feedback re-activates the PI3K-mammalian target of rapamycin (mTOR) pathway in tumors, resulting in compromised effectiveness of the PI3K inhibitors [34,35]. Using several model tumors in mice, Cantley and colleagues found that treatment with PI3K inhibitors causes systemic feedback that results in increased blood glucose and insulin [36]. This elevated glucose-insulin response activates the PI3K pathway, circumventing the action of the inhibitor. Based on this, the Cantley group examined approaches to limit the glucose-insulin feedback by placing mice bearing tumor allografts on a ketogenic diet, which is low-carbohydrate and high-fat and includes adequate protein [37], or by treating the mice with metformin or inhibitors of the sodium-glucose co-transporter 2 (SLGT2), in combination with PI3K inhibition. The Cantley group found that pre-treatment of the mice with metformin, prior to the introduction of a PI3K inhibitor, had a minimal effect on blood glucose and insulin elevation; however, the addition of SLGT2 inhibitors or placing the mice on a ketogenic diet reduced hyperglycemia, hyperinsulinemia, and signaling through the mTOR pathway [36]. A key finding of this work was that the addition of a ketogenic diet improved the drug efficacy of an array of agents that target the PI3K pathway. Importantly, a ketogenic diet alone was not sufficient for the observed effect and in some instances was more harmful when used in isolation [36]. Overall, the work presented by Professor Cantley suggests a paradigm shift in how cancer patients may be effectively treated with PI3K inhibitors by combining treatment protocols with a ketogenic diet to limit the unwanted glucose-insulin feedback. This could present a novel research avenue to be pursued in low-survival cancers with dependence on insulin or PI3K/Akt signaling [38].

## 6. Chromatin Reorganization by CTCF and CTCFL in Cancer Influences Aberrant Transcription

Research presented by Professors Jane Skok, the Sandra and Edward H. Meyer Professor of Radiation Oncology, Department of Pathology at New York University Lagone Health, focused on understanding the impact of a 3D chromatin organization on gene regulation in cancer using both wet and dry lab approaches. In particular, the Skok group was interested in a major structural protein involved in chromatin looping, the CCCTC-binding factor, CTCF. CTCF is a downstream target protein of growth-factor induced cell signaling pathways and is regulated by epidermal growth factor (EGF) and insulin through activation of PI3K-AKT and the mitogen-activated protein kinase/extracellular signal-regulated kinase (MAPK-ERK) signaling cascades [39]. The group is currently analyzing the impact of CTCF mutations associated with cancer [40,41]. Studies center on the impact of these mutations on: (i) CTCF binding affinity, (ii) binding site specificity, and (iii) their contribution to tumorigenicity. Ubiquitously expressed CTCF is involved in numerous cellular functions, such as organizing chromatin into self-interacting, topologically associated domains (TADs) that are important for gene regulation. Therefore, it is anticipated that some of these mutations could have a major biological impact. The mutations also provide an opportunity to study underlying CTCF-mediated gene regulation.

The Skok lab also studies the paralog of CTCF, namely CTCFL (CTCF-like). Normally, CTCFL is only expressed in the testis; however, it is also aberrantly expressed in many cancers. While it is known that shared and unique zinc finger sequences in CTCF and CTCFL enable CTCFL to bind competitively to a subset of CTCF binding sites, as well as its own unique locations, the impact of CTCFL on chromosome organization and gene expression has not been comprehensively analyzed in the context of CTCF function. Using an inducible complementation system, the Skok group analyzed the impact of expressing CTCFL and CTCF-CTCFL chimeric proteins in the presence or absence of endogenous CTCF to clarify the relative and combined contribution of CTCF and CTCFL to chromosome organization and transcription [42]. By analyzing CTCF and CTCFL binding in tandem, the group identified phenotypically distinct sites with respect to motifs, targeting promoter/intronic intergenic regions and chromatin folding. Finally, Professor Skok revealed that the N, C, and zinc finger terminal domains play unique roles in targeting each paralog to distinct binding sites to regulate transcription, chromatin looping, and insulation. Overall, the study presented by Professor Skok clarifies the unique and combined contribution of CTCF and CTCFL to chromosome organization and transcription, with direct implications for understanding how their co-expression deregulates transcription in cancer. Genetic profiling identifies both CTCF and CTCFL as being of relevance with regard for low-survival cancers, including ovarian, gastric, and esophageal cancers [41].

## 7. The Future of Low-Survival Cancer Research in Ireland

The IACR annual conference has historically had a strong focus on developing emerging talent and providing a platform for early-stage researchers to present their work. Seed funding and travel fellowship awards commenced in 2019. In 2020, the conference opened with the IACR/European Association for Cancer Research (EACR) Early Career Symposium and Workshop, a full day of events designed to educate and inform postgraduate and postdoctoral researchers. The conference program also contained dedicated sessions to postgraduate and postdoctoral researchers, including the Patrick G. Johnston Award for Excellence in Cancer Research Outreach, the Professor John Fitzpatrick Oral Poster Presentations, a proffered papers session dedicated to PhD and postdoctoral researchers, the Irish Cancer Society Biomedical Session, the EACR Junior and Senior Research Awards, and the Breakthrough Cancer Research Session, dedicated to research on low-survival cancers. This year, each early-stage researcher session had a strong representation of research on poor prognosis cancers. Approximately 10% of patients with a diagnosis of metastatic HER2+ breast achieved a durable complete response to therapy, but the majority do not [43]. Dr Neil Conlon, Dublin City University, received the EACR Junior Investigator Award for his work exploring the re-purposing of leukemia SRC/Abl tyrosine kinase inhibitor, dasatinib, for the treatment of treatment refractory HER2+ breast cancer in combination with the pan-HER tyrosine kinase inhibitor. As previously mentioned, subsets of colorectal cancer patients have poor outcomes, and this year, research on CRC featured. Dr Manuela Salvucci, Royal College of Surgeons in Ireland (RCSI), received the EACR Senior Investigator Award for her work using integrated multi-omics analyses from multi-region resections to uncover further detailed taxonomies for CRC. Dr Sudipto Das (RCSI) presented data showing that the novel DNA methylation landscape of metastatic CRC reveals the functional role of epigenetically regulated disease-associated enhancer regions, providing insight into potential epigenetic therapies and received the best proffered paper award for postdoctoral scientists. Amy McCorry (Queen’s University Belfast) presented on how antigen processing and presentation dictates prognosis in the fibroblast-rich subtype of stage II and III colon cancer to win the best PhD proffered paper. The Irish Cancer Society Biomedical session saw Dr Sophia Halliday, Queen’s University Belfast (QUB), presenting research on tumor immune cell gene expression signatures in prostate cancer and the relationship with clinical characteristics. Romina Silva, University College Dublin (UCD), presented her postgraduate studies on longitudinal analysis of personal DNA methylome patterns in metastatic prostate cancer. Pancreatic cancer has one the highest mortality rates (National Cancer Registry, Ireland). Better in vitro models are required to re-capitulate the pancreatic tumor microenvironment to identify novel clinically relevant therapies. Shannon Nelson, Dublin City University (DCU), presented her PhD studies on primary cell line organoids as a novel and alternative 3D organotypic cell model for pancreatic cancer and was awarded the Professor Patrick G. Johnston Award. The Professor John Fitzpatrick Oral Poster Presentation award went to Rachel Bleach for her work on levels of androstenedione in breast cancer patients whose disease recurs on aromatase inhibitor therapy. Jennifer Quinn, University College Cork (UCC), presented a proffered paper on the induction of autophagy following treatment with chemotherapeutic agents and its ability to promote recovery and chemo-resistance in ovarian cancer cells, winning the Breakthrough Cancer Research session award. The focus on low-survival cancers and cancer subtypes and the breadth of approaches employed by early-stage researchers displays the intent of Irish cancer research to tackle the research areas of greatest clinical need, now and in the future.

## 8. Conclusions

The conference concluded with the IACR Award for Outstanding Contribution to Cancer Medicine and Research, which went to Professor Joe (Michael J) Duffy, University College Dublin (UCD). Professor Duffy delivered a lecture entitled “Biomarkers and Therapeutic Targets in Breast Cancer: From Laboratory Research to Clinical Trials.” Professor Duffy’s work on the urokinase plasminogen activator (uPA) and plasminogen activator inhibitor-1 (PAI-1) has led to the development of one of the best validated prognostic biomarkers available for lymph node-negative breast cancer. Tumour tissue uPA and PAI-1 levels can be used to identify lymph node-negative patients with HER-2-negative tumors that can be safely spared adjuvant chemotherapy. Professor Duffy’s presentation highlighted the immense value of translational research for developing strategies to optimize patient treatment. Such biomarker approaches are critical to the progression of research on low survival rate cancers, where clinical subgroups can be identified to pursue the most appropriate research strategy. 

In recent years, the increased prominence of molecularly targeted agents and immune-based therapies has started to move cancer research towards more tumor agnostic, target-defined approaches. An advantage of this approach is that researchers are not limiting their work to a single cancer type but rather following the target or the system of interest, as exemplified by Professor Skok’s and Professor Cantley’s work. As each new targeted approach is developed, subgroups of low-survival cancers may benefit, making stepwise improvements for patient subpopulations. Historically, some cancer types have more limited profiles in relation to public awareness and the subsequent research funding available. Exciting new innovations, such as the first pre-habilitation exercise service for patients in a national cancer center in Ireland, presented by Sarah Moore, Clinical Specialist Physiotherapist, St. James Hospital, Dublin, Ireland, won the Nursing and Allied Health Sciences sessions proffered paper award. This service is already affecting patient response to gastro-intestinal and thoracic cancer surgery. It is clear from the 56th IACR Annual Conference that there is a vibrant research effort focusing on low-survival cancers in Ireland. The number of early-career researchers establishing their research expertise in this area bodes well for future findings that will improve outcomes for patients that currently have limited treatment options and survival outcomes.

## Appendix A

**Table A1:** Contributing speakers.

Speaker	Affiliation
Professor Helen Coleman	Cancer Epidemiology Research Group at the Centre for Public Health at Queen’s University Belfast (QUB), UK.
Dr. Aideen Ryan	The School of Medicine, Pharmacology and Therapeutics at the National University of Ireland-Galway (NUIG), Ireland
Professor Mary Helen Barcellos-Hoff	Department of Radiation Oncology, The University of California, San Francisco (UCSF), USA.
Professor Lewis Cantley	Sandra and Edward Meyer Cancer Center at Weill Cornell Medicine, New York, USA
Professor Jane Skok	Department of Pathology at New York University (NYU) Lagone Health, USA.
